# [^15^O]H_2_O PET/MRI for Assessment of Complete Response to Neoadjuvant or Induction Chemotherapy in Patients with Muscle-Invasive Bladder Cancer: A Pilot Study

**DOI:** 10.3390/jcm13164652

**Published:** 2024-08-08

**Authors:** Stefanie Korsgaard Körner, Lars Poulsen Tolbod, Bodil G. Pedersen, Thierry Boellaard, Rikke Vilsbøll Milling, Simone Buchardt Brandt, Mads Agerbæk, Lars Dyrskjøt, Kirsten Bouchelouche, Jørgen B. Jensen

**Affiliations:** 1 Department of Urology, Aarhus University Hospital, 8200 Aarhus, Denmarkbjerggaard@skejby.rm.dk (J.B.J.); 2 Department of Clinical Medicine, Aarhus University Hospital, 8200 Aarhus, Denmark; 3 Department of Radiology, Regional Hospital Horsens, 8700 Horsens, Denmark; 4 Department of Nuclear Medicine and PET, Aarhus University Hospital, 8200 Aarhus, Denmarkkirsten.bouchelouche@auh.rm.dk (K.B.); 5 Department of Radiology, Aarhus University Hospital, 8200 Aarhus, Denmark; 6 Department of Radiology, Netherlands Cancer Institute, Antoni van Leeuwenhoek Hospital, 1066 CX Amsterdam, The Netherlands; 7 Department of Oncology, Aarhus University Hospital, 8200 Aarhus, Denmark; 8 Department of Molecular Medicine, Aarhus University Hospital, 8200 Aarhus, Denmark

**Keywords:** bladder cancer, blood flow, magnetic resonance imaging, muscle invasive, neoadjuvant chemotherapy, perfusion, positron emission tomography

## Abstract

**Background:** Accurate assessment of therapy response to chemotherapy could possibly offer a bladder-sparing approach in selected patients with localized muscle-invasive bladder cancer (MIBC). The aim of this study was to evaluate whether [^15^O]H_2_O PET/MRI can be used for assessment of complete local pathological response to preoperative chemotherapy in patients with MIBC. **Methods:** This prospective pilot study included 13 patients with MIBC treated with neoadjuvant or induction chemotherapy and subsequent radical cystectomy. Patients underwent a [^15^O]H_2_O PET/MRI scan before chemotherapy and another scan after chemotherapy before radical cystectomy. Volumes of interest were delineated on T2-weighted MRI and transferred to parametric images for dynamic analysis. Tumor blood flow (TBF) was estimated by [^15^O]H_2_O PET. Changes in TBF were compared with histopathology. The Wilcoxon matched-pairs signed-ranks test was used for comparing pre- and post-chemotherapy measurements. **Results:** Mean TBF decreased by 49%. Mean TBF in complete responders (ypT0N0/ypTis) was not significantly different from non-complete responders (≥ypT1) (*p* = 0.52). **Conclusions:** Despite a measurable decrease in TBF after chemotherapy treatment, we were not able to estimate a TBF threshold for identifying complete responders to chemotherapy for MIBC patients. Further studies are needed to elucidate the potential of [^15^O]H_2_O PET/MRI in assessing therapy response in MIBC.

## 1. Introduction

At the time of diagnosis, approximately 25% of bladder cancer patients present with muscle-invasive bladder cancer (MIBC) [1]. The current guidelines on MIBC recommend treatment with cisplatin-based neoadjuvant chemotherapy and radical cystectomy in eligible patients [1]. Neoadjuvant chemotherapy treats micro metastatic disease and has been shown to improve overall survival by 5–8% at 5 years [2,3]. At time of cystectomy, up to 50% of patients treated with neoadjuvant chemotherapy have complete pathological response (ypT0N0) [4,5,6]. 

Accurate assessment of therapy response to chemotherapy could possibly offer a bladder-sparing approach in selected patients. However, no optimal method for evaluating therapy response prior to cystectomy has yet been established. 

Patients undergoing preoperative chemotherapy are currently evaluated by CT scanning before the last cycle of neoadjuvant chemotherapy or during induction chemotherapy to rule out development of new metastatic lesions, whereas studies have shown that CT has a limited role in local therapy assessment in bladder cancer [7,8]. 

Studies on multiparametric magnetic resonance imaging (MRI) for evaluating therapy response to neoadjuvant treatment are promising, especially for diffusion-weighted imaging (DWI) [9,10]. DWI seems to be superior to both T2-weighted sequences and dynamic contrast-enhanced imaging in assessing response to chemoradiotherapy [11]. Response to neoadjuvant chemotherapy has been shown to be associated with a significant increase in mean change in the apparent diffusion coefficient (ADC) compared with poor responders [10]. 

Another method for tumor evaluation is the quantification of tumor blood flow (TBF). ^15^O-labeled water is the gold standard for positron emission tomography (PET) quantification of regional tissue perfusion and for noninvasive imaging to quantify TBF [12,13]. [^15^O]H_2_O is an ideal flow tracer since it is freely diffusible and metabolically inert. [^15^O]H_2_O PET has been used for TBF measurements in different types of cancer: brain, head and neck, and prostate [14,15]. TBF measurements as an assessment of treatment response to neoadjuvant chemotherapy seems possible in breast cancer [16,17]. 

The purpose of the current study was to evaluate whether [^15^O]H_2_O PET/MRI could assess local therapy response to chemotherapy in patients with MIBC and estimate a TBF threshold for identifying patients with complete pathological local response. 

## 2. Materials and Methods

### 2.1. Inclusion

Patients diagnosed with cT2-T4aN0-N1M0 bladder cancer who were fit for cisplatin-based neoadjuvant or induction chemotherapy and subsequent radical cystectomy were eligible for the study. Clinical TNM stage was evaluated by transurethral resection of the bladder tumor (TURBT), CT-urography, and ^18^F-fluordeoxyglucose (FDG) PET-CT. 

Exclusion criteria were contra-indications for MRI or hip alloplastic replacement ipsilateral to the bladder tumor. For patients with clinical N0, four cycles of neoadjuvant chemotherapy were recommended, whereas for patients with clinical N1, six cycles of induction chemotherapy were recommended. Patients who received <3 cycles of neoadjuvant or induction chemotherapy were excluded from the study.

### 2.2. Imaging 

**Figure 1:**
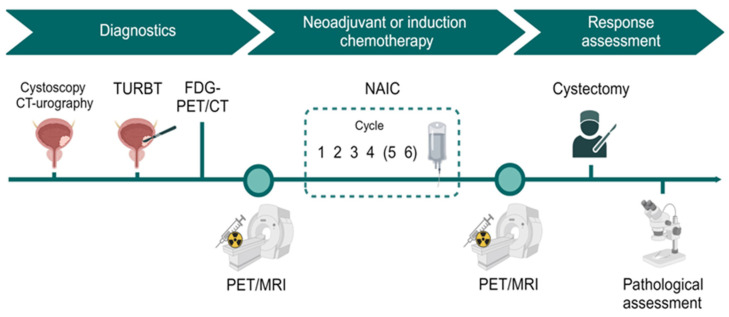
Timeline of study. TURBT = transurethral resection of bladder tumor; NAIC = neoadjuvant or induction chemotherapy. Created in biorender.com.

Study participants underwent [^15^O]H_2_O PET/MRI before the start of chemotherapy but after TURBT. After completion of ≥3 cycles of neoadjuvant or induction chemotherapy, patients underwent a second [^15^O]H_2_O PET/MRI scan before cystectomy (Figure 1).

All [^15^O]H_2_O PET/MRI scans were performed on a 3.0 Tesla Magnet PET/MRI scanner (GE Signa PET/MR, GE Healthcare, Milwaukee, WI, USA). All patients fasted for four hours prior to the scan. Patients were asked to void 30 min before the scan and to drink 200 mL of water to ensure bladder filling. To reduce bowel movement, an intramuscular injection of glucagon (GlucaGen 1 mg, Novo Nordisk A/S, Bagsværd, Denmark) was given shortly before the start of the scan. 

#### 2.2.1. MRI

MRI scans were performed with 4 mm T2-weighted images (T2w) of the pelvis in the sagittal, coronal, and axial plane. Diffusion-weighted imaging (DWI) of the pelvis (b-values 50, 400, 800 s/mm^2^) was obtained, and ADC map and b = 1400 s/mm^2^ images were calculated. T2w MRI (3 mm) and DWI were obtained perpendicular to the tumor-bearing part of the bladder wall for optimal tumor tissue representation for TBF measurements. No contrast-enhanced sequences were obtained.

#### 2.2.2. PET

**Figure 2:**
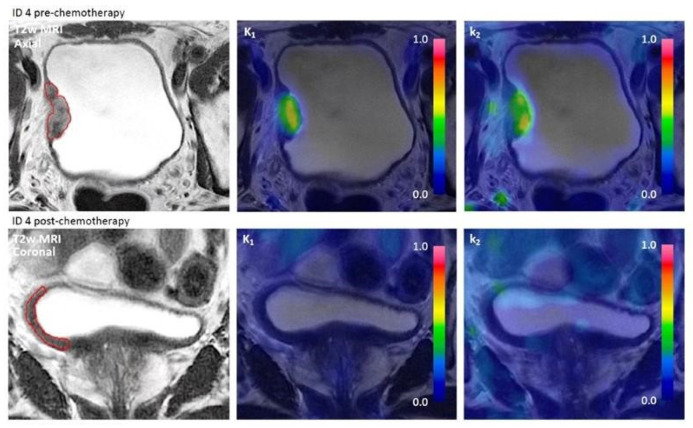

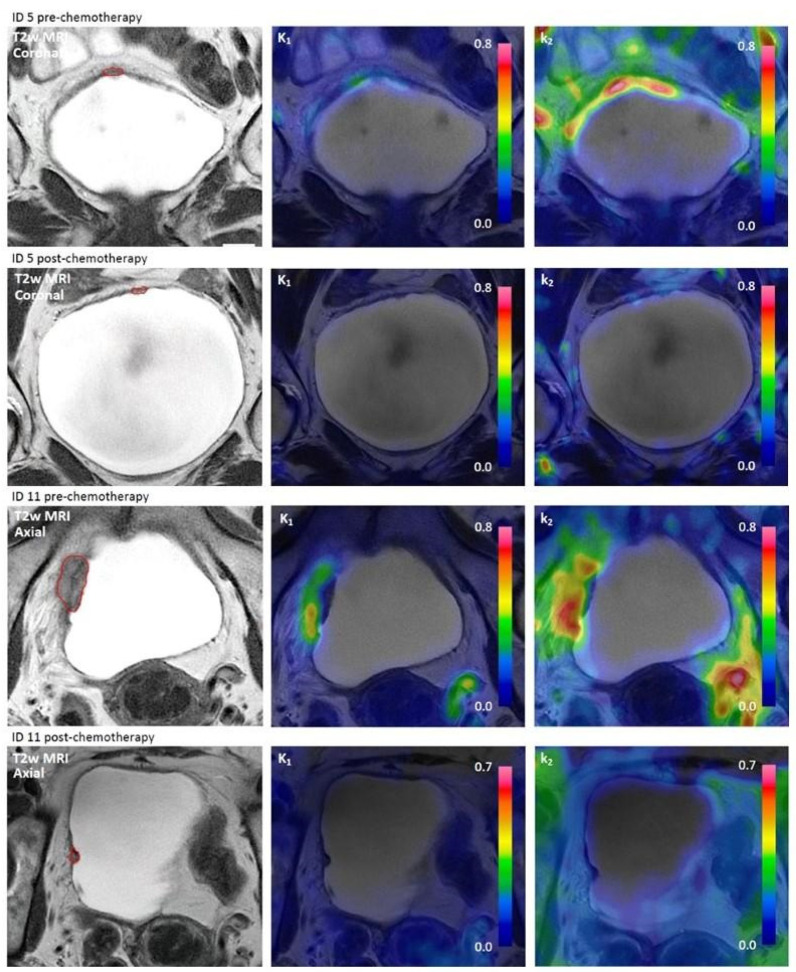
T2-weighted (T2w) MRI and [^15^O]H_2_O PET parametric images (K_1_ and k_2_) pre- and post-chemotherapy of three study patients. ID 4 had wall thickening on T2w MRI but decreased tumor blood flow (TBF) post-chemotherapy and had ypT1b + Tis. ID 5 had no visible tumor and decreased TBF post-chemotherapy and had ypT0. ID 11 had suspect volume of interest but decreased TBF post-chemotherapy, and patient had ypT0.

Two PET scans were performed per patient per session: a pelvic scan and a cardiac scan. Firstly, a scan of the pelvis was performed, and then a scan of the heart for the image-derived input function. The duration of both scans was 6 min and the scans started at the same time that a bolus injection of 400 MBq [^15^O]H_2_O (5–10 mL) was administered in a peripheral vein. The bolus injection was standardized (20 mL at 1 mL/s followed by a flush of 35 mL saline at 2 mL/s) to ensure consistency between the two scans using a contrast infusion pump (MedRad MRXperion, Bayer Medical Care, Indianola, PA, USA). [^15^O]H_2_O PET and MRI of the bladder were performed simultaneously (Figure 2). 

### 2.3. Image Analysis

MRI served two purposes: (i) to delineate tumor VOI on MRI for dynamic analysis after fusion with PET and (ii) to evaluate complete local therapy response in the bladder by morphological changes in MRI sequences. 

MRI analysis was performed by a radiologist with 15 years of specialization in pelvic MRI and the primary investigator. The tumor was localized on T2w MRI by the primary investigator in consensus with the radiologist and tumor VOI was manually delineated. Tumor VOIs were defined as areas suggestive of residual tumor on T2w MRI, evaluated by the presence of an intermediate signal (relatively hyperintense compared with the bladder wall muscle). Tumor identification on DWI and T2w sequences were evaluated for correspondence regarding location. Restricted diffusion on DWI (b = 1400) with a corresponding low signal on the ADC map indicated residual tumor. Tumor VOI was manually delineated on DWI and mapped to the corresponding ADC map to ensure the location when registering ADC values for tumor VOI. The single lowest ADC value and mean ADC value of tumor VOI were registered. 

Tumor identification on post-chemotherapy MRI was performed by two radiologists with 5 and 15 years of specialization in pelvic MRI, blinded to the histopathology of the cystectomy specimen. T2w MRI and DWI were evaluated separately for presence of residual tumor and a dichotomous response (complete tumor response/near-complete tumor response or residual tumor) was registered. In the case of one or two dubious pixels on DWI, the response ‘near-complete tumor response’ was used. The presence of an intermediate signal on T2w MRI was marked as a tumor VOI suggestive of residual tumor. Hyperintense spots on DWI b-1400 with a corresponding low signal on the ADC map were found to indicate residual tumor. The single ADC value and mean ADC value of tumor VOI on post-chemotherapy scans were registered. 

VOIs were manually delineated on the 3 mm T2w MRI by visual guidance and transferred to the dynamic PET series for TBF measurements using a Hermes Hybrid Viewer or Hermes Affinity Viewer (Hermes Medical Solutions, Stockholm, Sweden). 

### 2.4. Dynamic Analysis

Time–activity curves (TACs) for the tumor were extracted from the VOIs. An image-derived input function was used by cluster analysis of both the pelvic scan and the cardiac scan. The pelvic image-derived input function was used to correct the cardiac image-derived input function for delay and dispersion [15]. A one-tissue compartment model was used for kinetic modeling, where K_1_ reflects mean TBF in all of the tumor and k_2_ reflects mean TBF in the perfused part of the tumor [15]. For the remainder of the manuscript, K_1_ is used as the measure of TBF. The perfusable tissue fraction (PTF) was calculated as K_1_/k_2_. 

### 2.5. Pathologic Evaluation

Histopathological findings were reported according to the 8th edition of the TNM classification [18]. In the case of uncertainty regarding residual bladder tumor at the first pathologic routine investigation, an additional evaluation was performed with pan cytokeratin AE1/AE3 immunohistochemistry and additional step sectioning for hematoxylin and eosin staining of the previous tumor area/ulcerated mucosa/retracted areas to find any microscopic residual tumor cells. 

### 2.6. Statistical Analysis 

Complete tumor response (CTR) was defined as either no tumor and no detectable tumor cells identified in the cystectomy specimen by histopathological evaluation (ypT0N0) or carcinoma in situ (ypTis) in the specimen. Patients were stratified according to persistent muscle-invasive tumor (ypT2-T4) at the time of cystectomy or no persistent muscle-invasive tumor (ypT0-T1/Tis). 

Cohen’s kappa statistics (κ, 95% confidence interval (CI)) were used to estimate inter-rater reliability, raw percent agreement, and agreement between the radiologist and histopathology of the cystectomy specimen. 

The Wilcoxon matched-pairs signed-ranks test was used when comparing the following pre- and post-chemotherapy measurements: single lowest ADC value, mean ADC value, TBF, and PTF. For comparison of the change in PTF, one patient (ID 6) was excluded in this analysis due to extreme outliers. 

To consider any possible confounding influence of tumor size on perfusion parameters, the Spearman’s rank correlation coefficient was used to assess the relationship between tumor size and TBF.

Statistical tests were two-sided with the significance level set at *p* < 0.05. Study data were collected and managed using the REDCap (Vanderbilt University Medical Center, Nashville, TN, USA) electronic data capture tool, hosted at Aarhus University [19]. Data analysis was performed using R software (version 4.2.3; R Foundation for Statistical Computing, Vienna, Austria) [20]. 

## 3. Results

### 3.1. Patient and Tumor Characteristics

**Figure 3:**
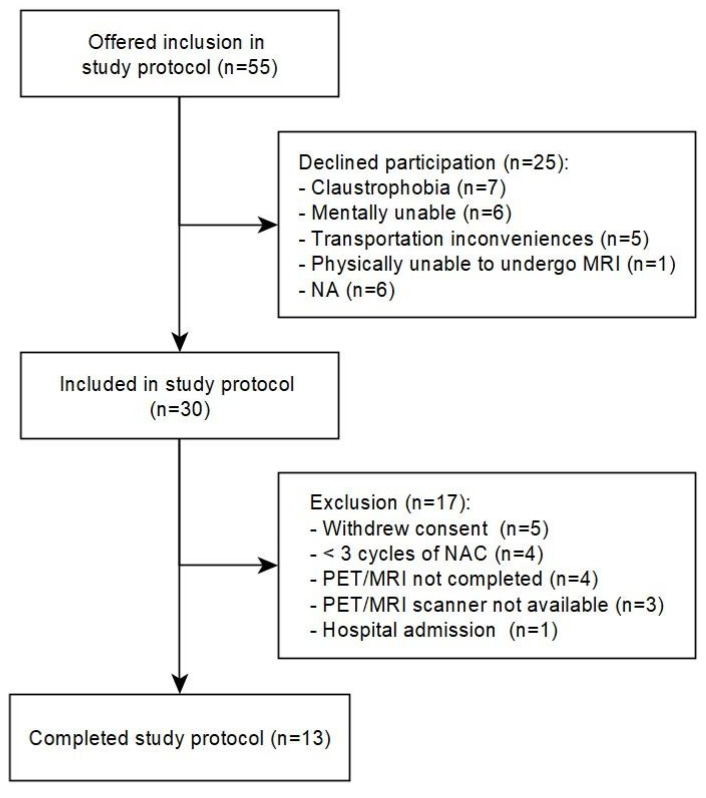
Flowchart of inclusion.

Between June 2020 and June 2023, 55 patients diagnosed with MIBC and scheduled for neoadjuvant or induction chemotherapy and radical cystectomy at a single university hospital center were offered inclusion in the study protocol. Of these, 30 patients were enrolled in the study and 13 patients (43%) completed a [^15^O]H_2_O PET/MRI scan before and after chemotherapy (Figure 3). Table 1 shows the patient, clinical, and histopathological details of the 13 study patients who completed protocol. The median age was 64 years (range 50–72 years). The median time from study enrollment to radical cystectomy was 123 days (range 85–180 days). The median time from TURBT to pre-chemotherapy PET/MRI was 32 days (range 20–65 days). The median time from last dose of chemotherapy to post-chemotherapy PET/MRI scan was 19 days (range 11–34 days). 

All patients were assessed as at minimum cT2 with proven muscle invasion in the TURBT specimen by histopathological evaluation. Three patients had urothelial carcinoma with variant histology and one patient had a neuroendocrine bladder tumor and was treated with four cycles of cisplatin and etoposide. Two patients had cN1 disease and were treated with six cycles of gemcitabine and cisplatin. 

Six patients had ypT0N0 by histopathological evaluation (Table 1). Two patients had ypTis. Five patients had residual tumor and three of these had ≥ypT2. In cases of more than one tumor, the largest tumor was measured and used for TBF measurement. Ten patients had a solitary tumor (Supplementary Table S1). 

### 3.2. MRI Findings and Interrater Reliability

Seven patients were evaluated as having residual tumor on either T2w MRI or DWI. Six patients were evaluated as having no residual tumor on both T2w MRI and DWI (Supplementary Table S2). 

Neither single lowest ADC value nor mean ADC value of tumor VOI had any additional clinical value in predicting complete tumor response (Supplementary Figure S1).

The Cohen’s kappa inter-rater reliability and raw percent agreement were κ = 0.52 (95 CI: 0.10;0.94, *p* = 0.02) and 77% for T2w imaging and κ = 0.84 (95% CI: 0.55;1.00, *p* < 0.001) and 92% for DWI, respectively. The agreement between histopathology of the cystectomy specimen and reader 1 and reader 2 was κ = −0.21 (95% CI: −0.72;0.30, *p* = 0.42) and κ = −0.10 (95% CI: −0.63;0.44, *p* = 0.72), respectively (Supplementary Table S3). Sensitivity, specificity, positive predictive value, and negative predictive value for MRI assessment by each reader are given in Supplementary Table S4. No residual tumor on both MRI and DWI assessment was used versus complete response for pathological evaluation of the cystectomy specimen.

### 3.3. Relationship of Tumor Blood Flow and Tumor Size

**Figure 4:**
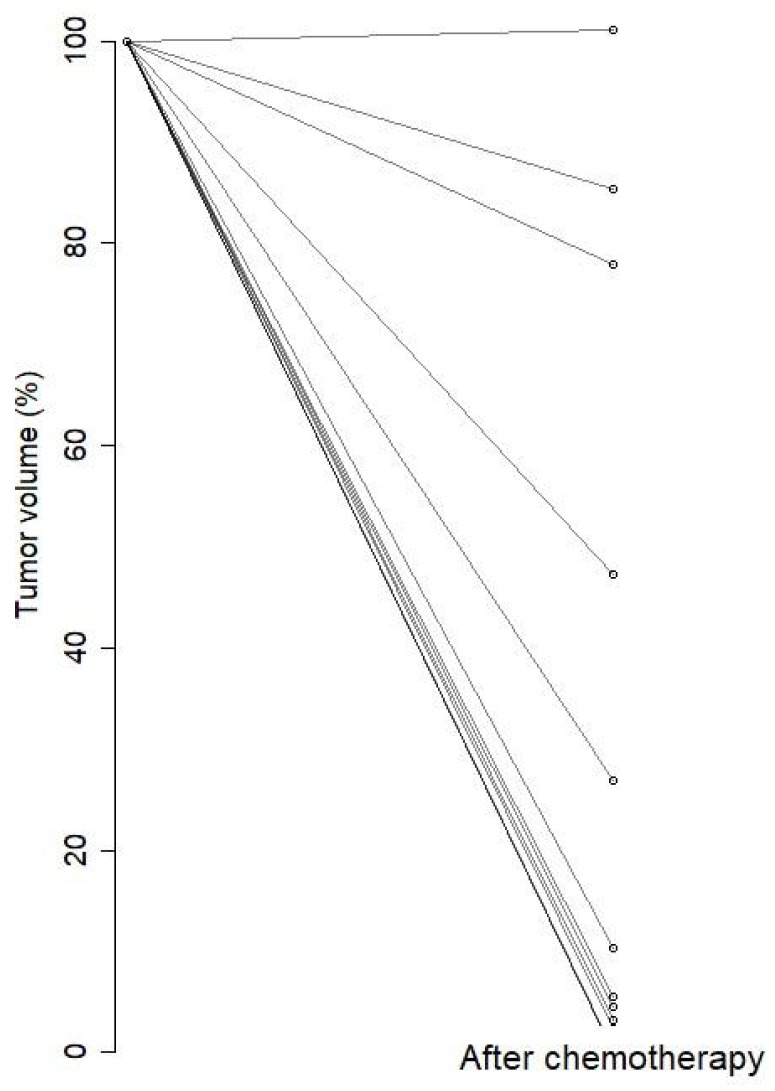
Percentage change in tumor volume from pre-chemotherapy to post-chemotherapy MRI.

Figure 4 shows the percentage change in tumor size post-chemotherapy when tumor size pre-chemotherapy is set at 100%. We found no statistically significant correlation between pre-chemotherapy TBF and spherical tumor volume at pre-chemotherapy PET/MRI (rho = 0.05, *p* = 0.86), either when evaluating the association between change before and after chemotherapy in tumor volume and change in TBF (rho = 0.08, *p* = 0.79). 

### 3.4. Tumor Blood Flow

For all patients, the median TBF was 0.33 (IQR: 0.16–0.40) mL/min/mL before chemotherapy and 0.10 (IQR: 0.08–0.14) mL/min/mL after chemotherapy (Figure 4). A statistically significant difference was found between the pre-chemotherapy TBF and the post-chemotherapy TBF (*p* = 0.0046). The mean TBF decreased by 49%. For complete tumor responders and non-complete tumor responders, the mean decline in TBF was 63% and 27%, respectively. The difference in decreased mean TBF for complete tumor responders (*n* = 8) and for non-complete tumor responders (*n* = 5) was non-significant (*p* = 0.52). When stratifying according to complete tumor response or not, a statistically significant difference was found for patients with complete tumor response between pre- and post-chemotherapy values for TBF (*p* = 0.0078) (Supplementary Figure S2A). Similar results were found for TBF when stratifying for persistent muscle-invasive tumor or not with statistically significant difference for patients with no persistent muscle-invasive tumor (*p* = 0.014) (Supplementary Figure S2B).

**Figure 5:**
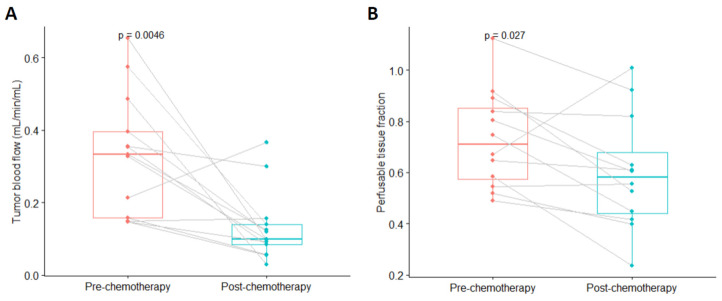
Tumor blood flow and perfusable tissue fraction pre- and post-chemotherapy. (**A**) Tumor blood flow. (**B**) Perfusable tissue fraction.

When comparing PTF pre- and post-chemotherapy for all patients, we found a statistically significant difference (*p* = 0.027) (Figure 5B). When stratifying PTF for clinical outcome (persistent muscle-invasive tumor or not and complete tumor response or not) we found no statistically significant difference between pre-chemotherapy and post-chemotherapy PTF (Supplementary Figure S3). 

## 4. Discussion

Our study examined whether [^15^O]H_2_O PET/MRI could assess local response to preoperative chemotherapy in patients with MIBC. We found that the mean TBF decreased by 49%. However, the mean decrease in TBF was not significantly different in patients with complete tumor response compared to patients with non-complete tumor response. Moreover, we were not able to estimate a TBF threshold for identifying complete tumor responders to chemotherapy. 

Several studies have evaluated the repeatability coefficient in [^15^O]H_2_O PET studies for other types of cancer. Lubberink et al. and van der Veldt et al. found a repeatability coefficient for TBF in liver lesions of 28% and in lung cancer of 20%, respectively [21,22]. According to De Langen et al., TBF changes of more than 18% are likely to represent treatment effect in lung cancer [23]. For non-complete tumor responders, we found a mean TBF reduction of 27%. 

Yoshida et al. reported that even though DWI was superior to T2w, DWI still missed microscopic residual cancer [11]. The issue of microscopic residual cancer missed on MRI is also reported in the present study. We identified three cases where both radiologists evaluated T2w and DWI as complete/near-complete tumor response but residual tumor was reported by histopathologic evaluation. In two of these cases, the pathological evaluation reported no macroscopic residual cancer. However, one case had ypT2b in a 1 mm focus and one case had ypT1b by microscopic histopathological evaluation. The third case had T1a in a 2 mm focus. Both radiologists evaluated patient ID 6 as having complete/near-complete tumor response on T2w and DWI. In our analysis, we regarded carcinoma in situ (CIS) as complete tumor response since we did not expect MRI or PET to be able to detect CIS. 

Inflammatory or fibrotic changes related to transurethral resection or chemotherapy treatment complicate the radiological assessment of treatment response on T2w. Bladder wall thickening complicates therapy response assessment on T2w. This could result in overestimation of VOI on post-chemotherapy T2w when ensuring delineation of the tumor suspect area for dynamic analysis. It must be assumed that VOI delineation is overestimated in patients without clearly visible residual tumor on MRI, especially on the post-chemotherapy scan, to ensure inclusion of areas with possible residual tumor. For example, ID 6 and ID 9 had no obvious solid tumor on MRI, and volume was interpreted as TURBT sequelae. However, as areas of bladder wall thickening might still contain residual tumor, volume was included in the analyses. Therefore, it is recommended to perform MRI ideally prior to TURBT or at least 2 weeks after for more accurate assessment [24]. In our study, patients were included at the time of diagnosis of MIBC, and MRI was therefore performed after TURBT. 

The measured decrease in TBF post-chemotherapy could be due to either local response to chemotherapy or time since TURBT, depending on whether the pre-chemotherapy scan showed increased TBF due to inflammatory changes from the TURBT. The pre-chemotherapy scan was performed 20–65 days after TURBT. For ID 5, Figure 2, increased perfusion on the pre-chemotherapy parametric image could resemble sequelae after TURBT; however, PET/MRI was performed 47 days after TURBT, which should be adequate time for healing of the mucosa. For ID 11, the pre-chemotherapy scan was performed 21 days after TURBT and increased perfusion might partly reflect TURBT sequelae. 

When interpreting the inter-rater reliability as suggested by McHugh, the level of agreement was weak for T2w but strong for DWI [25]. To improve inter-rater reliability, standardized imaging interpretation protocols should be performed and radiologists should undergo additional training. We saw negative agreement between both readers and histopathology, reflecting that MRI sequences were unable to predict tumor response. 

This present study is a small pilot study from a single institution, which limits the conclusions that can be drawn from the study. However, this study is the first to quantify TBF in bladder cancer by [^15^O]H_2_O PET/MRI. Apart from the small numbers, other limitations include that our study population was heterogeneous and included two patients with N+ disease and one third of our patients had variant histology. [^15^O]H_2_O PET/MRI is an expensive imaging method compared to a computed tomography scan; however, by using an imaging method for assessing therapy response, patients could possibly avoid invasive diagnostic methods. Micro-ultrasound could be another promising non-invasive imaging modality but further studies are needed [26]. Possible future novel techniques should improve clinical management and this should be highlighted in future studies. 

In future, combination analyses including circulating tumor DNA and urine tumor DNA detecting of minimal residual disease might be an additional aid in selecting patients for a bladder-sparing approach. 

## 5. Conclusions 

In this first pilot study on [^15^O]H_2_O PET/MRI in MIBC patients, we were able to detect a decrease in tumor blood flow following chemotherapy. However, we were not able to establish a tumor blood flow threshold able to differentiate patients with complete tumor response to preoperative chemotherapy from patients with residual tumor in the bladder. Further studies are needed to elucidate the potential of [^15^O]H_2_O PET/MRI in assessing therapy response in bladder cancer. 

## Figures and Tables

**Table 1:** Patient characteristics and clinicopathological details.

Case	Patients		cTNM Stage	Histological Subtype	Spherical Tumor Volume Pre-Treatment (cm^3^)	Change in Spherical Tumor Volume	NAIC Cycles	CTH Regimen	ypTNM Stage at RC
	Age	Sex							
1	58	M	cT2N1M0	UC	0.8	−95%	6	GC	ypT4aN1M0 + Tis
2	68	F	cT3N1M0	UC	0.2	−53%	6	GC	ypT2bN2M1
3	52	M	cT2N0M0	UC	0.4	−100%	4	GC	ypT0N0M0
4	65	M	cT3N0M0	NET	3.8	−100%	4	CE	ypT1bN0M0 + Tis
5	59	F	cT2N0M0	UC + SD	0.2	−15%	3	GC	ypT0N0M0
6	64	M	cT2N0M0	UC	10.2 *	−90%	3	GC	ypTisN0M0
7	72	M	cT3bN0M0	UC	43.1	−95%	3	GC	ypT1aN0M0
8	69	M	cT2N0M0	UC	0.6	+1%	4	GC	ypT0N0M0
9	50	M	cT2N0M0	UC + SD	7.1 *	−22%	4	GC	ypT0N0M0
10	68	M	cT2N0M0	UC + SD	0.4	−73%	4	GC	ypT2bN0M0
11	61	F	cT3N0M0	UC	5.2	−97%	4	GC	ypT0N0M0
12	72	M	cT2N0M0	UC	2.8	−98%	4	GC	ypT0N0M0
13	54	M	cT2N0M0	UC	11.4	−100%	4	GC	ypTisN0M0

UC = urothelial carcinoma; SD = squamous differentiation; NET = neuroendocrine tumor; NAIC = neoadjuvant or induction chemotherapy; CTH = chemotherapy; RC = radical cystectomy; GC = gemcitabine and cisplatin; CE = cisplatin and etoposide. * Volume is TURBT sequelae, no obvious solid tumor.

## Data Availability

The data presented in this study are available on request from the corresponding author. The data are not publicly available due to privacy restrictions.

## Supplementary Materials

The following supporting information can be downloaded at: https://www.mdpi.com/article/10.3390/jcm13164652/s1, Figure S1: ADC-values of tumor VOI pre- and post-chemotherapy, Table S1: Tumor characteristics and measurements pre- and post-treatment, Figure S2: Tumor blood flow pre- and post-chemotherapy, Table S2: Radiologist assessment of MRI sequences, Figure S3: Perfusable tissue fraction pre- and post-chemotherapy, Table S3: Inter-rater reliability for MRI sequences and between reader and pathology, Table S4: Sensitivity, specificity, positive predictive value, and negative predictive value for MRI assessment by reader 1 and reader 2.

## References

[1] Witjes J.A., Bruins H.M., Cathomas R., Compérat E.M., Cowan N.C., Gakis G., Hernández V., Linares Espinós E., Lorch A., Neuzillet Y. (2020). European Association of Urology Guidelines on Muscle-invasive and Metastatic Bladder Cancer: Summary of the 2020 Guidelines. Eur. Urol..

[2] Yin M., Joshi M., Meijer R.P., Glantz M., Holder S., Harvey H.A., Kaag M., van de Putte E.E.F., Horenblas S., Drabick J.J. (2016). Neoadjuvant Chemotherapy for Muscle-Invasive Bladder Cancer: A Systematic Review and Two-Step Meta-Analysis. Oncologist.

[3] Vale C.L. (2005). Neoadjuvant chemotherapy in invasive bladder cancer: Update of a systematic review and meta-analysis of individual patient data advanced bladder cancer (ABC) meta-analysis collaboration. Eur. Urol..

[4] Grossman H.B., Natale R.B., Tangen C.M., Speights V.O., Vogelzang N.J., Trump D.L., deVere White R.W., Sarosdy M.F., Wood D.P., Raghavan D. (2003). Neoadjuvant chemotherapy plus cystectomy compared with cystectomy alone for locally advanced bladder cancer. N. Engl. J. Med..

[5] Pfister C., Gravis G., Fléchon A., Chevreau C., Mahammedi H., Laguerre B., Guillot A., Joly F., Soulié M., Allory Y. (2022). Dose-Dense Methotrexate, Vinblastine, Doxorubicin, and Cisplatin or Gemcitabine and Cisplatin as Perioperative Chemotherapy for Patients With Nonmetastatic Muscle-Invasive Bladder Cancer: Results of the GETUG-AFU V05 VESPER Trial. J. Clin. Oncol..

[6] Körner S.K., Jensen J.B. (2021). A population-based retrospective analysis on variation in use of neoadjuvant chemotherapy depending on comorbidity in patients with muscle-invasive bladder cancer undergoing cystectomy in Denmark in the period 2013–2019. Scand. J. Urol..

[7] Mogos H., Eriksson E., Styrke J., Sherif A. (2020). Computerized tomography before the final treatment cycle of neoadjuvant chemotherapy or induction chemotherapy in muscle- invasive urinary bladder cancer, cannot predict pathoanatomical outcomes and does not reflect prognosis—Results of a single centre re. Transl. Androl. Urol..

[8] Fukui T., Matsui Y., Umeoka S., Inoue T., Kamba T., Togashi K., Ogawa O., Kobayashi T. (2016). Predictive value of radiological response rate for pathological response to neoadjuvant chemotherapy and post-cystectomy survival of bladder urothelial cancer. Jpn. J. Clin. Oncol..

[9] Necchi A., Bandini M., Calareso G., Raggi D., Pederzoli F., Farè E., Colecchia M., Marandino L., Bianchi M., Gallina A. (2020). Multiparametric Magnetic Resonance Imaging as a Noninvasive Assessment of Tumor Response to Neoadjuvant Pembrolizumab in Muscle-invasive Bladder Cancer: Preliminary Findings from the PURE-01 Study. Eur. Urol..

[10] Hafeez S., Koh M., Jones K., Ghzal A.E., D’Arcy J., Kumar P., Khoo V., Lalondrelle S., McDonald F., Thompson A. (2022). Diffusion-weighted MRI to determine response and long-term clinical outcomes in muscle-invasive bladder cancer following neoadjuvant chemotherapy. Front. Oncol..

[11] Yoshida S., Koga F., Kawakami S., Ishii C., Tanaka H., Numao N., Sakai Y., Saito K., Masuda H., Fujii Y. (2010). Initial experience of diffusion-weighted magnetic resonance imaging to assess therapeutic response to induction chemoradiotherapy against muscle-invasive bladder cancer. Urology.

[12] Frackowiak R.S., Lenzi G.L., Jones T., Heather J.D. (1980). Quantitative measurement of regional cerebral blood flow and oxygen metabolism in man using 15O and positron emission tomography: Theory, procedure, and normal values. J. Comput. Assist. Tomogr..

[13] Herscovitch P., Markham J., Raichle M.E. (1983). Brain blood flow measured with intravenous H2(15)O. I. Theory and error analysis. J. Nucl. Med..

[14] Lehtio K., Eskola O., Viljanen T., Oikonen V., Gronroos T., Sillanmaki L., Grenman R., Minn H. (2004). Imaging perfusion and hypoxia with PET to predict radiotherapy response in head-and-neck cancer. Int. J. Radiat. Oncol. Biol. Phys..

[15] Tolbod L.P., Nielsen M.M., Pedersen B.G., Hoyer S., Harms H.J., Borre M., Borghammer P., Bouchelouche K., Frokiaer J., Sorensen J. (2018). Non-invasive quantification of tumor blood flow in prostate cancer using (15)O-H2O PET/CT. Am. J. Nucl. Med. Mol. Imaging.

[16] Mankoff D.A., Dunnwald L.K., Gralow J.R., Ellis G.K., Schubert E.K., Tseng J., Lawton T.J., Linden H.M., Livingston R.B. (2003). Changes in blood flow and metabolism in locally advanced breast cancer treated with neoadjuvant chemotherapy. J. Nucl. Med..

[17] Dunnwald L.K., Gralow J.R., Ellis G.K., Livingston R.B., Linden H.M., Specht J.M., Doot R.K., Lawton T.J., Barlow W.E., Kurland B.F. (2008). Tumor metabolism and blood flow changes by positron emission tomography: Relation to survival in patients treated with neoadjuvant chemotherapy for locally advanced breast cancer. J. Clin. Oncol..

[18] Brierley J., Gospodarowicz M., Wittekind C. (2017). TNM Classification of Malignant Tumours.

[19] Harris P.A., Taylor R., Thielke R., Payne J., Gonzalez N., Conde J.G. (2009). Research electronic data capture (REDCap)—A metadata-driven methodology and workflow process for providing translational research informatics support. J. Biomed. Inform..

[20] R Core Team (2020). R: A Language and Environment for Statistical Computing.

[21] Lubberink M., Golla S.S., Jonasson M., Rubin K., Glimelius B., Sörensen J., Nygren P. (2015). ^15^O-Water PET Study of the Effect of Imatinib, a Selective Platelet-Derived Growth Factor Receptor Inhibitor, Versus Anakinra, an IL-1R Antagonist, on Water-Perfusable Tissue Fraction in Colorectal Cancer Metastases. J. Nucl. Med..

[22] Van Der Veldt A.A.M., Hendrikse N.H., Harms H.J., Comans E.F.I., Postmus P.E., Smit E.F., Lammertsma A.A., Lubberink M. (2010). Quantitative Parametric Perfusion Images Using ^15^O-Labeled Water and a Clinical PET/CT Scanner: Test–Retest Variability in Lung Cancer. J. Nucl. Med..

[23] De Langen A.J., Lubberink M., Boellaard R., Spreeuwenberg M.D., Smit E.F., Hoekstra O.S., Lammertsma A.A. (2008). Reproducibility of Tumor Perfusion Measurements Using ^15^O-Labeled Water and PET. J. Nucl. Med..

[24] Panebianco V., Narumi Y., Altun E., Bochner B.H., Efstathiou J.A., Hafeez S., Huddart R., Kennish S., Lerner S., Montironi R. (2018). Multiparametric Magnetic Resonance Imaging for Bladder Cancer: Development of VI-RADS (Vesical Imaging-Reporting And Data System). Eur. Urol..

[25] McHugh M.L. (2012). Interrater reliability: The kappa statistic. Biochem. Med..

[26] Calace F.P., Napolitano L., Arcaniolo D., Stizzo M., Barone B., Crocetto F., Olivetta M., Amicuzi U., Cirillo L., Rubinacci A. (2022). Micro-Ultrasound in the Diagnosis and Staging of Prostate and Bladder Cancer: A Comprehensive Review. Medicina.

