# Supplementary material for: [15O]H2O PET/MRI for Assessment of Complete Response to Neoadjuvant or Induction Chemotherapy in Patients with Muscle-Invasive Bladder Cancer: A Pilot Study
Source: J Clin Med. 2024 Aug 8;13(16):4652. doi: 10.3390/jcm13164652 (PMC11354727; doi:10.3390/jcm13164652)
Supplement: Supplementary file 1 [file jcm-13-04652-s001.zip › jcm-3057609-supplementary.pdf]

Supplementary Table S1. Tumor characteristics and measurements pre- and post-treatment.

Case	Number of tumors	Largest tumor diameter pre-treatment (cm)	Largest tumor diameter post-treatment (cm)	Single ADC-value pre-treatment (10^{-6} mm^2/s)	Single ADC-value post-treatment (10^{-6} mm^2/s)	Mean ADC-value pre-treatment (10^{-6} mm^2/s)	Mean ADC-value post-treatment (10^{-6} mm^2/s)
1	1	1.89	0.60	559	500	847	963
2	1	1.31	0.62	206	847	1090	847
3	3	1.50	0	476	638	1318	630
4	1	2.01	0	298	454	647	NA
5	1	0.84	0.75	100	294	208	294
6	2	4.04*	1.20†	158	547	672	694
7	1	5.90	0.66	182	772	525	699
8	1	2.09	2.42	578	1400	1008	702
9	3	4.88*	3.45†	331	697	906	858
10	1	1.12	2.06†	376	850	673	470
11	1	3.02	0.67	375	851	633	851
12	1	3.51	0.60	352	465	667	723
13	1	4.07	0.32	395	774	912	991

* diameter is TURBT sequelae, no obvious solid tumor.
† measurement of bladder wall thickening.

Supplementary Table S2. Radiologist assessment of MRI sequences.

	**Reader 1**		**Reader 2**		**Pathologic evaluation at RC**	
ID	T2w	DWI	T2w	DWI	ypTNM stage	Macroscopic residual tumor
1	-	+	+	+	ypT4aN1M0 + Tis	No
2	-*	+	+	+	ypT2bN2M1	No
3	-	+	+	+	ypT0N0M0	No
4	-	-	-	-	ypT1bN0M0 + Tis	No
5	+	+	+	+	ypT0N0M0	No
6	-	-	-	-	ypTisN0M0	Yes
7	-	-	-	-	ypT1aN0M0	Yes
8	-	+	-	-	ypT0N0M0	No
9	+	-	+	-	ypT0N0M0	No

10	-	-	-	-	ypT2bN0M0	No
11	-	-	-	-	ypT0N0M0	No
12	+	+	+	+	ypT0N0M0	No
13	-	-	-	-	ypTisN0M0	Yes

T2w = T2-weighted imaging; DWI = diffusion-weighted imaging; RC = radical cystectomy.
+ = residual tumor; - = complete tumor response/near-complete tumor response.
*no residual tumor in bladder but visually malignant suspect lymph nodes.
Green color marks agreement between readers.

Supplementary Table S3. Interrater reliability for MRI sequences and between reader and pathology.

MRI		Pathology	
**T2w**	κ = 0.52 (95 CI: 0.10;0.94, p = 0.02)	**Reader 1**	κ = -0.21 (95% CI: -0.72;0.30, p = 0.42)
**DWI**	κ = 0.84 (95% CI: 0.55;1.00, p < 0.001)	**Reader 2**	κ = -0.10 (95% CI: -0.63;0.44, p = 0.72)

Supplementary Table S4. Sensitivity, Specificity, positive predictive value, and negative predictive value for reader 1 and reader 2.

	**Reader 1**	**Reader 2**
Sensitivity	0.40	0.40
Specificity	0.38	0.50
Positive predictive value	0.29	0.33
Negative predictive value	0.50	0.57

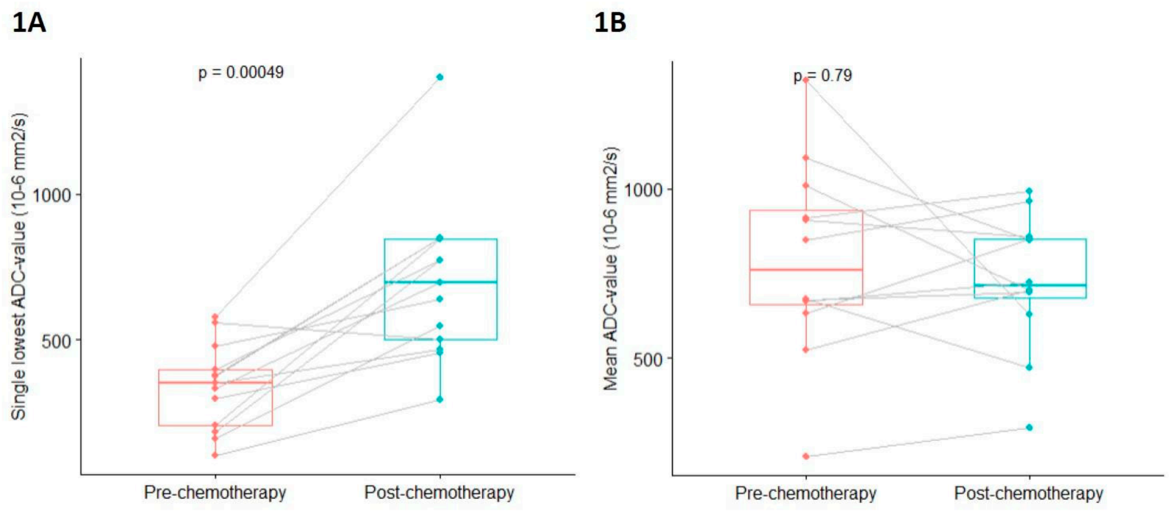

Supplementary Figure S1. ADC-values of tumor VOI pre- and post-chemotherapy. (1A) Single lowest ADC-value evalueted on DWI. (1B) Mean ADC-value.

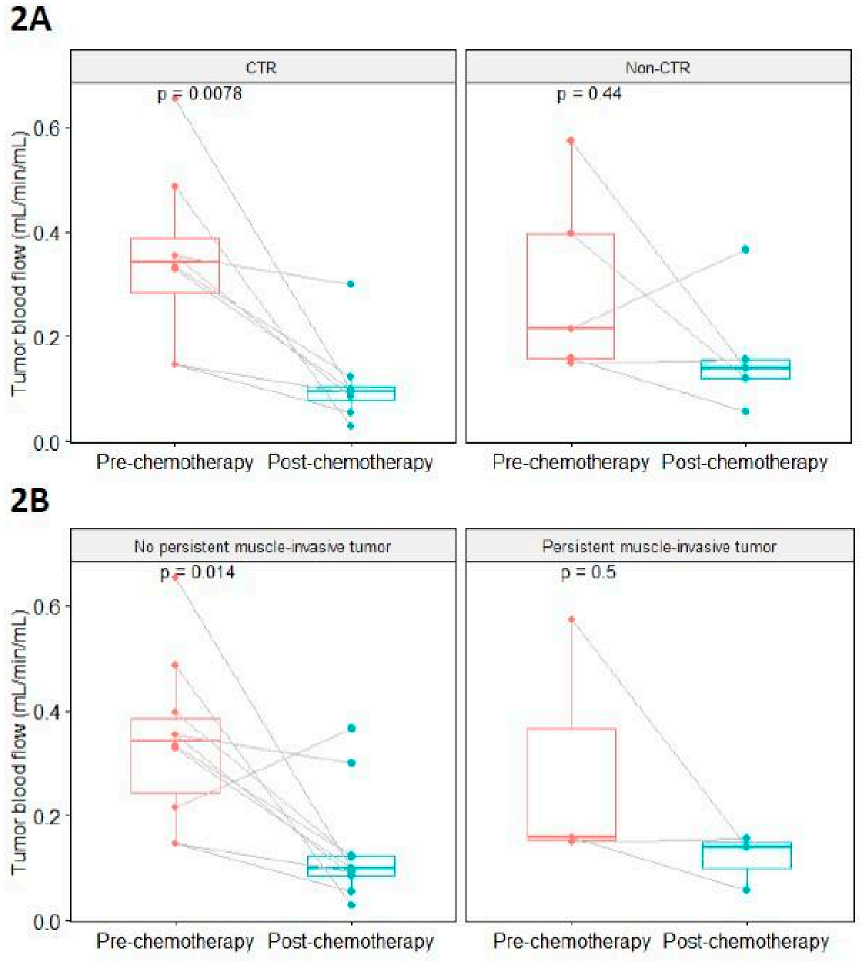

Supplementary Figure S2. Tumor blood flow pre- and post-chemotherapy. (2A) Patients stratified according to complete tumor response (CTR: ypT0NO/ypTis) in cystectomy specimen or not. (2B) Patients stratified according to muscle-invasive tumor in cystectomy specimen or no muscle-invasive tumor.

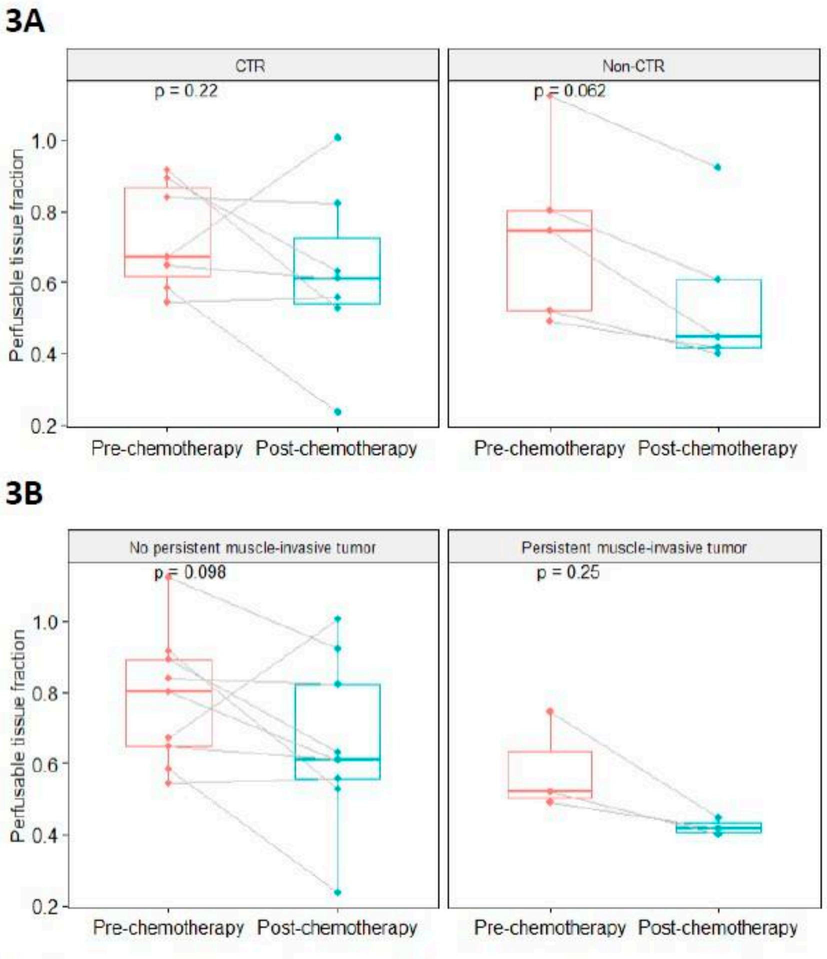

Supplementary Figure S3. Pefusable tissue fraction pre- and post- chemotherapy. (3A) Patients stratified according to complete tumor response (CTR: ypT0N0/ypTis) in cystectomy specimen or not. (3B) Patients stratified according to muscle-invasive tumor in cystectomy specimen or no muscle-invasive tumor.
